# Exploitation of the Sick

**Published:** 1914-10

**Authors:** 


Exploitation of the Sick.—In a recent issue of *Public Health Reports* reference is made to the fact that from time to time there have appeared upon the market in the Southern States preparations advertised as cures for pellagra. These preparations have usually sold at prices that would be exorbitant even for remedies of value in the treatment of the disease. When one considers that most pellagra patients are found among people of small means and frequently of comparatively little experience and education, the advantage taken of the sick and unfortunate by the manufacturers of these nostrums appears in its true light.

The Public Health Service has examined several of these preparations. Those examined were received put up in packages containing usually a bottle of liquid and a box of tablets or capsules, sufficient in amount to last the patient for a short time (two to four weeks), and were marked to sell, some for $5 and one for $10 a package. The preparations upon examination were found to contain inexpensive inorganic salts, such as iron, magnesium, lime, and sulphur. One consisted mainly of copperas, charcoal, and small amounts of quinine.

Nothing was found in these preparations which, so far as the scientific world has been able to learn through the laborious investigations of trained workers, has any value in the treatment of pellagra. Some of the ingredients might be of service at times to relieve some of the symptoms. On the other hand, some of the ingredients would undoubtedly aggravate other symptoms, so that these preparations on the whole are probably not only not beneficial but really do harm to the sick.

Pellagra is a disease in which the patient has times when he is quite ill, followed in many cases by periods of weeks, maybe months, when he feels comparativey well. Then, too, some pellagra patients get better regardless of the drugs or medicines they may take. Among those who have taken the nostrums above referred to are, naturally, patients who have improved, some who have got well, and some who have died, the same as among pellagrins who have not taken these preparations. Owing to the natural tendency to give credit for receovery to that medicine or drug which one happens to be taking, a number of pellagrins undoubtedly sincerely believe they have been helped by the taking of these nostrums, with the result that the manufacturers have no difficulty in obtaining testimonials as to their beneficial effects.

